# Expression of activator protein-1 (AP-1) family members in breast cancer

**DOI:** 10.1186/1471-2407-13-441

**Published:** 2013-09-28

**Authors:** Amirhossein Razavirad, Hui Gao, Reza Ghiasvand, Chunyan Zhao, Kazem Zendehdel, Karin Dahlman-Wright

**Affiliations:** 1 Department of Biosciences and Nutrition, Karolinska Institutet, Stockholm, S-14183 Huddinge Sweden; 2 Cancer Research Center, Cancer Institute of Iran, Tehran University of Medical Sciences, Tehran, Iran; 3 Department of Biostatistics, Institute of Basic Medical Sciences, University of Oslo, Oslo, Norway

**Keywords:** AP-1 family members, Breast cancer, Estrogen receptor, Progesterone receptor

## Abstract

**Background:**

The activator protein-1 (AP-1) transcription factor is believed to be important in tumorigenesis and altered AP-1 activity was associated with cell transformation. We aimed to assess the potential role of AP-1 family members as novel biomarkers in breast cancer.

**Methods:**

We studied the expression of AP-1 members at the mRNA level in 72 primary breast tumors and 37 adjacent non-tumor tissues and evaluated its correlation with clinicopathological parameters including estrogen receptor (ER), progesterone receptor (PR) and HER2/neu status. Expression levels of Ubiquitin C (UBC) were used for normalization. Protein expression of AP-1 members was assessed using Western blot analysis in a subset of tumors. We used student’s t-test, one-way ANOVA, logistic regression and Pearson’s correlation coefficient for statistical analyses.

**Results:**

We found significant differences in the expression of AP-1 family members between tumor and adjacent non-tumor tissues for all AP-1 family members except Fos B. Fra-1, Fra-2, Jun-B and Jun-D mRNA levels were significantly higher in tumors compared to adjacent non-tumor tissues (*p* < 0.001), whilst c-Fos and c-Jun mRNA levels were significantly lower in tumors compared with adjacent non-tumor tissues (*p* < 0.001). In addition, Jun-B overexpression had outstanding discrimination ability to differentiate tumor tissues from adjacent non-tumor tissues as determined by ROC curve analysis. Moreover, Fra-1 was significantly overexpressed in the tumors biochemically classified as ERα negative (*p* = 0.012) and PR negative (*p* = 0.037). Interestingly, Fra-1 expression was significantly higher in triple-negative tumors compared with luminal carcinomas (*p* = 0.01).

**Conclusions:**

Expression levels of Fra-1 and Jun-B might be possible biomarkers for prognosis of breast cancer.

**Electronic supplementary material:**

The online version of this article (doi:10.1186/1471-2407-13-441) contains supplementary material, which is available to authorized users.

## Background

Breast cancer is the second most common cancer and the most common cancer among women in the world [1]. In addition, breast cancer is a heterogeneous disease that includes several distinct subtypes with distinctive gene expression patterns and different overall survival [2].

The treatment of breast cancer has been greatly advanced in the past decades due to the discovery of specific predictive and prognostic biomarkers that enable the application of more individualized therapies to different molecular subgroups with distinct clinical behavior [3]. Among the established biomarkers for breast cancer, estrogen receptor (ER) is the most powerful predictive marker both in determining prognosis and predicting response to hormone therapies [4]. Recently, human epidermal growth factor receptor 2 (HER2) has also become a routine marker in breast cancer predicting response to HER2 targeted therapy [5–7]. However, there is clearly a need to identify additional biomarkers for breast cancer as all breast cancers do not express ER and/or HER2 and additionally, there is no perfect correlation between these biomarkers and the response to targeted treatment.

It is well established that estrogen signaling and ERs play a central role in the development of breast cancer [8, 9]. Although many of the known effects of estrogen are mediated via a direct interaction of estrogen with ERs, ERα and ERβ, which regulate the expression of specific sets of genes through a direct interaction with cis-regulatory elements, estrogen-response elements (EREs), of target genes [9, 10], it is also well established that ERs interact with DNA indirectly through interaction with other DNA-bound transcription factors such as activator protein-1 (AP-1) complexes [11].

The AP-1 transcription factor is a dimeric complex that includes members of the JUN and FOS protein families. Unlike the JUN family members (c-Jun, Jun-B, Jun-D), the FOS family members (c-Fos, Fra-1, Fra-2 and Fos-B) need to hetrodimerize with members of the JUN family to form transcriptionally active complexes. After dimerization, AP-1 complexes bind to response elements on DNA including TPA response elements (TREs) and cAMP response elements (CREs) in the promoter and enhancer regions of target genes [12, 13]. *In vitro* studies have shown that FOS-JUN heterodimers form more stable complexes and can display stronger DNA-binding activity compared with JUN homodimers [12, 14].

Several studies investigated the expression of FOS and JUN family members at the mRNA and protein levels in breast cancer and suggested a role for these proteins as potential biomarkers in breast cancer [14–18]. However, a systemic evaluation of the expression of all AP-1 family members as potential biomarkers in breast cancer is still lacking.

In the present study we focused on the expression of c-Fos, Fra-1, Fra-2, Fos-B, c-Jun, Jun-B and Jun-D in human breast cancer tumors and adjacent non-tumor tissues with the aim to assay the potential of these molecules as novel biomarkers. Their correlation with ER status, progesterone receptor (PR) status, HER2 status, lymph node involvement, stage and grade was further investigated.

## Methods

### Tissue collection and tumor specimens

Tissue samples of 72 primary breast cancer specimens (mean age 48.6 years, median age 46.5 years; range 24- 85 years) and 37 adjacent non-tumor tissues were available. For 36 cases, paired samples from tumor and adjacent non-tumor tissues were available. Histologically all tumors were classified as invasive ductal and lobular carcinomas. ER, PR and HER2 statuses were available in 70, 62 and 68 cases and were positive in 47, 35 and 14 cases, respectively (Table 1). Receptor status was assessed using Immunohistochemistry (IHC). Fifty-two of the primary breast tumors were lymph node positive and 20 were lymph node negative. Thirty-eight patients were premenopausal and 32 postmenopausal, and for two patients the menopausal status was not available. Forty-two tumors classified as luminal (ER positive and/or PR positive, and HER2 negative), 10 as triple-negative (ER negative, PR negative and HER2 negative) and 14 as HER2-enriched (HER2 positive) (Table 1). The pathological staging was done as recommended by the American Joint Committee on Cancer (AJCC) TNM system. Eight tumors were classified as stage I, 37 as stage II, 25 as stage III and 2 as stage IV. Moreover, 25 patients classified as grade 1, 40 as grade 2, 6 as grade 3 and one as missing. All samples have been provided from the National Tumor Bank of the Cancer Institute of Iran. Informed consent was obtained from all patients who donated samples to the tumor bank. The National Research Ethics Committee of I.R of Iran and the Regional Research Ethics committee of Karolinska Institute approved the study.

**Table 1:** **Clinicopathological data**

**ER status**	
Positive (%)	47 (65.3)
Negative (%)	18 (25.0)
Weak (%)	5 (6.9)
Unknown (%)	2 (2.8)
**PR status**	
Positive (%)	35 (48.6)
Negative (%)	25 (34.7)
Weak (%)	2 (2.8)
Unknown (%)	10 (13.9)
**HER2 status**	
Positive (%)	14 (19.4)
Negative (%)	54 (75.0)
Weak (%)	2 (2.8)
Unknown (%)	2 (2.8)
**Subtypes of breast cancer**	
Luminal carcinomas (%)	42 (63.6)
Triple-Negative tumors (%)	10 (15.2)
HER2-enriched tumors (%)	14 (21.2)

### Real-time PCR analysis

RNA was extracted from fresh frozen tissues using RNeasy plus Universal Mini Kits (QIAGEN) according to the manufacturer’s instructions. The integrity and concentration of the RNA was assessed using the Agilent Bioanalyzer. Complementary DNA (cDNA) was synthesized using Superscript III First-Strand Synthesis SuperMix (Invitrogen), according to the manufacturer’s instructions. One μg RNA from each sample was used as starting material for cDNA synthesis.

Real-time PCR was run in triplicate in a 7500 ABI real-time PCR thermocycler (Applied Biosystems). ERα (ESR1), c-Fos and c-Jun mRNA expression were determined by TaqMan assay (Hs00174860_s1), TaqMan assay (Hs04194186_s1) and TaqMan assay (Hs01103582_s1), respectively. The ubiquitin C TaqMan assay (Hs00824723_m1) was used for normalization. The final volume per well for TaqMan assays was 15 μl. SYBR Green assays were used to determine the mRNA expression for Fra-1 (forward primer: GGA GGA AGG AAC TGA and reverse primer: CAC CAA CAT GAA CTC), Fra-2 (forward primer: AAG CTG CAG GCG GAG and reverse primer: CAC CAA CAT GAA CTC), Fos-B (forward primer: GAA CGA AAT AAA CTA and reverse primer: TTT TCT TCC TCC AAC), Jun-B (forward primer: CGC CGA CGG CTT TGT and reverse primer: GGT GTC ACG TGG TTC), Jun-D (forward Primer: CCA GCG AGG AGC AGG and reverse primer: GCT GGT TCT GCT TGT). The final volume per well for SYBR Green assays was 10 μl. The thermal cycling conditions were 95°C for 20 seconds once, then repetitively 95°C for 3 seconds and 60°C for 30 seconds for all assays.

The expression of 16 candidate endogenous control genes was analyzed by real-time PCR using the TaqMan Endogenous Control Assay on 16 randomly selected samples including 11 tumors and 5 adjacent non-tumor tissues to identify an optimal gene for normalization. The results showed that ubiquitin C (UBC) displayed the most stable expression among the samples and it was chosen for normalization (data not shown). The mRNA expression was calculated using the ΔCt method by subtracting the average Ct-value of triplicates of selected genes from the average Ct-value of triplicates of the housekeeping gene (UBC) as an internal control.

### Western blot analysis

Frozen tumor tissue was minced and cells were lysed with RIPA/complete mini lysis buffer. Protein extracts were prepared as described previously [14]. Forty μg of protein extract was analyzed by Western blot using c-Fos polyclonal antibody (H-125) sc-7202 (1:200), Fra-1 polyclonal antibody (R-20) sc-605 (1:400), Fra-2 polyclonal antibody (Q-20) sc-604 (1:800), Fos-B polyclonal antibody (102) sc-48 (1:200), c-Jun polyclonal antibody (H-79) sc-1694 (1:400), Jun-B monoclonal antibody (C-11) sc-8051 (1:100) and Jun-D polyclonal antibody (329) sc-74 (1:400). All antibodies were purchased from Santa Cruz biotechnology.

### Statistical analysis

Student’s t-test was performed to compare continuous variables between two different categorical clinicopathological characteristics including ERα, PR, HER2, lymph node status, and menopausal status. In addition, one-way ANOVA was used when comparing a continuous variable with several categorical explanatory variables such as subtypes of breast cancer, staging and BSR grading system. Curve estimation regression and logistic regression models were also fitted with the mRNA expression of AP-1 family members as the outcome variable.

ROC (Receiving Operating Characteristic) curve test was used and the area under the curve (AUC) was calculated to summarize and present the discrimination between tumor and adjacent non-tumor tissues, as the area under the curve defined previously [19]. An arbitrary level of 5% for statistical significance (two-sided) was considered in all analyses. Statistical analysis was calculated by means of SPSS statistical software version 16 and R software.

## Results

### Differential expression for AP-1 family members in tumors compared to adjacent tissues

**Figure 1:**
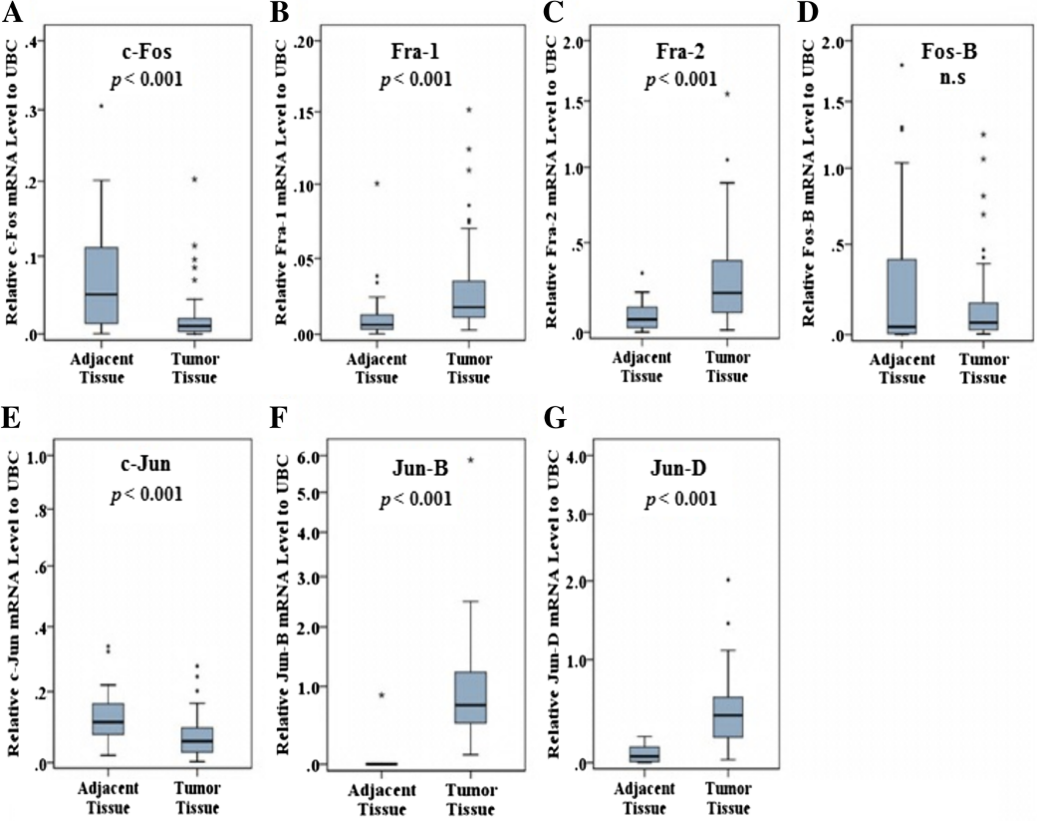
**Expression of AP-1 family members comparing tumor and adjacent tissues.** The expression of Fra-1 **(B)**, Fra-2 **(C)**, Jun-B **(F)** and Jun-D **(G)** are significantly higher in tumors compared with adjacent tissues (*p* < 0.001), whereas the expression of c-Fos **(A)** and c-Jun **(E)** are significantly lower in tumors compared with adjacent tissues (*p* < 0.001). A non-paired model was applied. Gene expression (y-axis) was quantified by real-time PCR and normalized to UBC.

The mRNA expression levels of AP-1 family members in 72 mammary carcinomas and 37 adjacent non-tumor tissues are shown in Figure 1.

Fra-1, Fra-2, Jun-B and Jun-D exhibited significantly higher expression in tumors compared with adjacent tissues (*p* < 0.001). C-Fos and c-Jun mRNA levels were significantly lower in tumors compared with adjacent tissues (*p* < 0.001). Finally, the expression of Fos-B did not differ between tumor tissue and adjacent tissue. Repeating the analysis using a paired design, for the 36 individuals where paired samples were available, produced similar results for the differences in expression levels between tumor tissue and adjacent tissue (Additional file 1: Figure S1).

**Figure 2:**
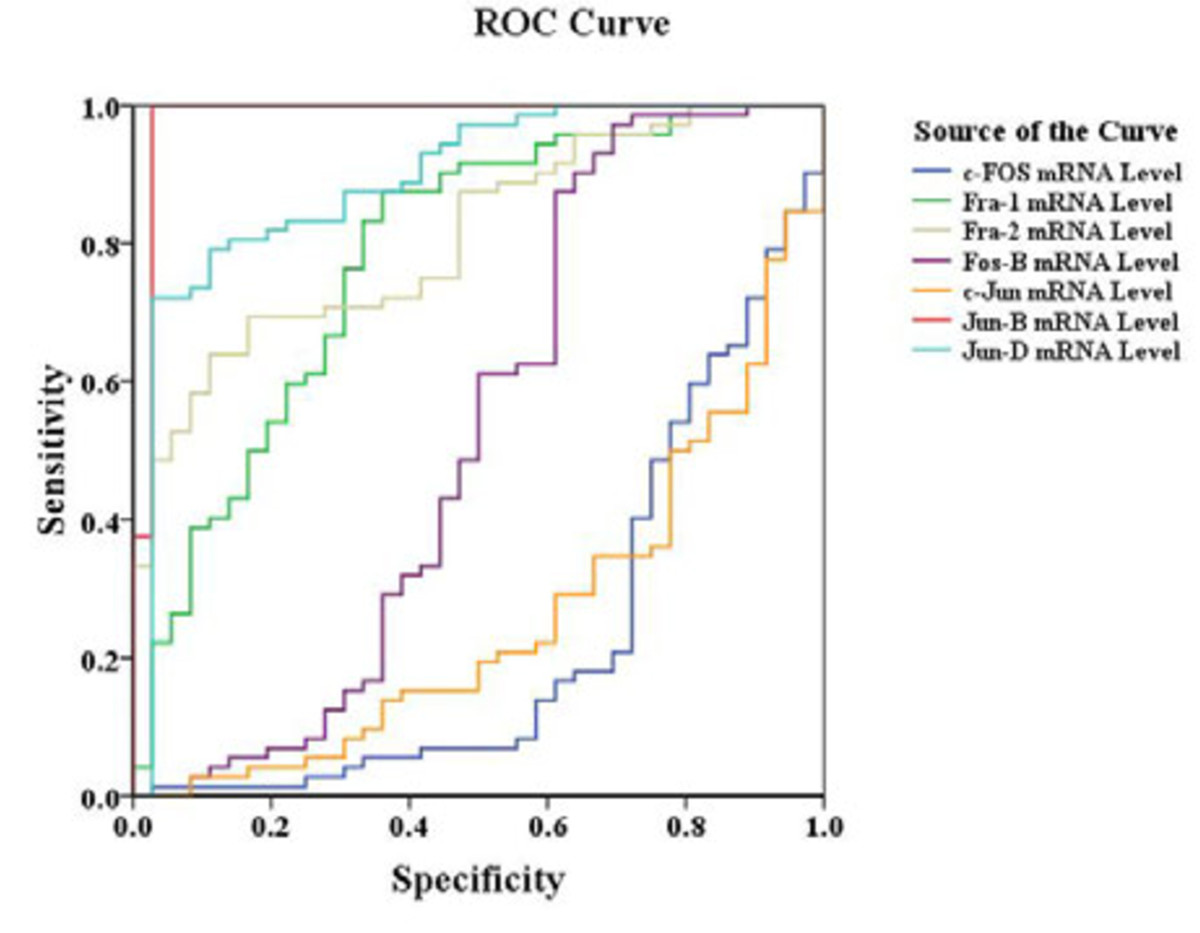
**ROC analyses comparing tumor and adjacent tissues.** Jun-B mRNA overexpression had outstanding discrimination ability (AUC = 0.983), and Jun-D, Fra-2 and Fra-1 had an acceptable discrimination ability.

ROC analysis showed that Jun-B overexpression had outstanding discrimination ability to differentiate tumor tissue from adjacent non-tumor tissue (AUC = 0.983) (Figure 2). In addition, Jun-D, Fra-2 and Fra-1 had acceptable discrimination abilities (AUC = 0.894, 0.811 and 0.782, respectively) (Table 2).

**Table 2:** **Area Under the Curve (AUC) of ROC analysis**

Test result variable(s)	AUC
**Jun-B** mRNA Level	0.983
**Jun-D** mRNA Level	0.894
**Fra-2** mRNA Level	0.811
**Fra-1** mRNA Level	0.782
**Fos-B** mRNA Level	0.522
**c-Jun** mRNA Level	0.258
**c-Fos** mRNA Level	0.230

The minimum value of AUC is 0.5; the maximum is 1. Jun-B overexpression has outstanding discrimination ability, and also Jun-D, Fra-2 and Fra-1 have acceptable discrimination abilities.

**Figure 3:**
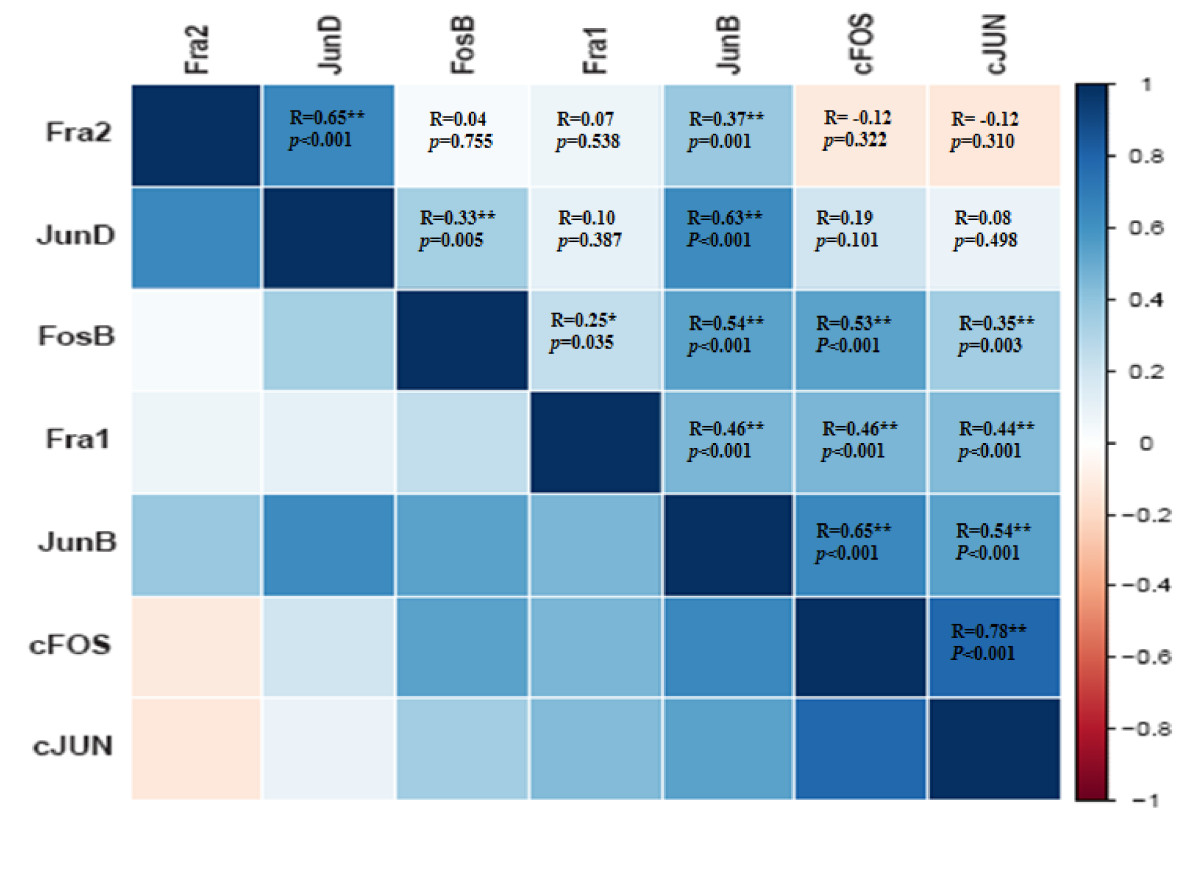
**Heat map of the Pearson’s correlation matrix for the mRNA expression of AP-1 members.** Pairwise correlation analyses were performed for all assayed mRNAs. Blue represents positive correlation for a given gene pair, and red represents negative correlation. C-Jun and c-Fos showed strong positive correlation (r = 0.78). In addition, there were moderate positive correlations between Jun-B and Jun-D (r = 0.63), and between Fra-2 and Jun-D (r = 0.65).

Pearson’s correlation analysis was performed pairwise for all seven AP-1 family members the expressions of which were analyzed in this cross sectional study (Figure 3). In this analysis, c-Jun and c-Fos showed strong positive correlation (r = 0.78). In addition, there were moderate positive correlations between Jun-B and Jun-D (r = 0.63), and between Fra-2 and Jun-D (r = 0.65). Furthermore, Jun-D and Jun-B displayed moderate positive correlation to ERα mNRA levels in ERα positive tumors (n = 47) (Additional file 1: Figure S2A and S2B).

### Association of mRNA expression levels with protein expression levels

**Figure 4:**
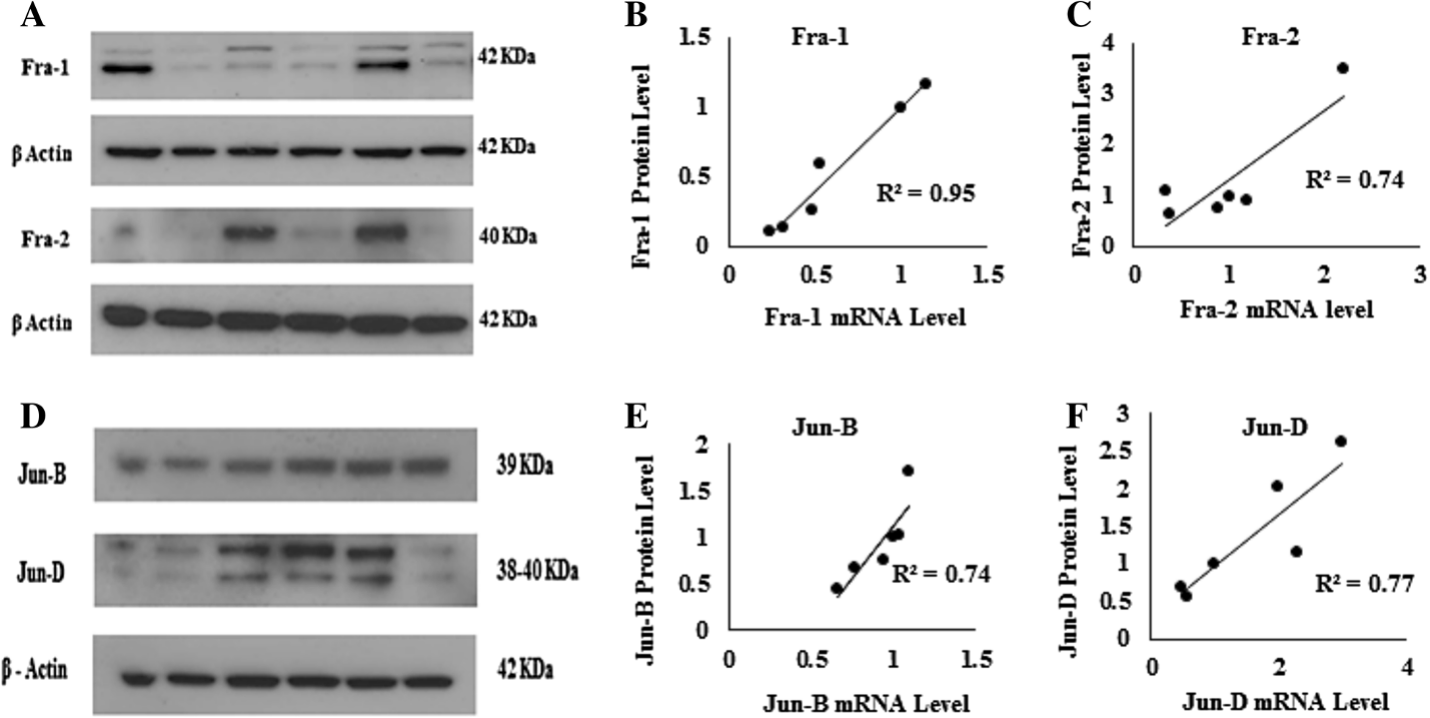
**Western blot analyses showing consistency between protein and mRNA expression levels in breast tumors.** Equal amounts of protein (40 μg) from 6 tumor tissues were loaded. β-actin was used as loading control. For Fra-1 and Jun-D, two bands with apparent molecular weights of approximately 40 and 42 KDa, and approximately 38 and 40 KDa were observed, respectively. Figures 4**A** and 4**D** show the Western blot analysis for Fra-1, Fra-2, Jun-B and Jun-D, respectively. The results of protein expression levels were compared with the corresponding mRNA expression levels showing good correlation between mRNA and protein expression levels for Fra-1 (R^2^ = 0.95), Fra-2 (R^2^ = 0.74), Jun-B (R^2^ = 0.74) and Jun-D (R^2^ = 0.77). Figure 4**B**,**C**,**E** and **F** show correlations between mRNA and protein expression levels for Fra-1, Fra-2, Jun-B and Jun-D, respectively.

Western blot analysis on six tumor samples demonstrated correlation between mRNA and protein levels for Fra-1, Fra-2, Jun-B and Jun-D (Figure 4). Figure 4A and D show the Western blot analysis for Fra-1, Fra-2, Jun-B and Jun-D, respectively. Figure 4B,C,E and F show correlations between mRNA and protein expression levels for Fra-1, Fra-2, Jun-B and Jun-D, respectively. The samples for these analyses were chosen based on that they displayed differential expression of AP-1 family members to allow correlation between mRNA expression levels and protein levels. The protein level of c-Jun did not perfectly correlate with mRNA expression level (data not shown). For c-Fos and Fos-B, protein expression was undetectable presumably due to low expression as we could detect positive controls for these proteins in the assays (data not shown).

### Fra-1 is overexpressed in triple-negative breast tumors

**Figure 5:**
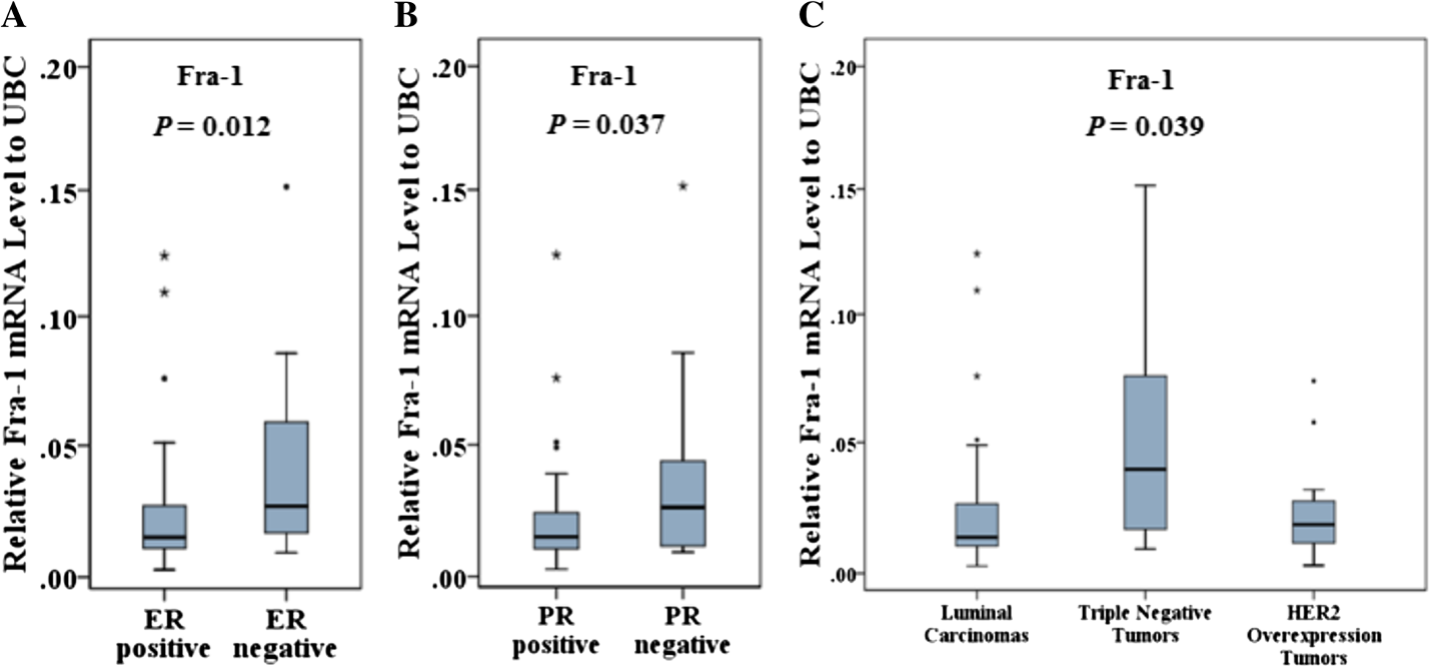
**mRNA expression of Fra-1 in relation to ER status, PR status and breast cancer subtypes.** The expression of Fra-1 is higher in tumors classified as ER negative and PR negative tumors compared with ER and PR positive tumors (*p* = 0.012 and *p* = 0.037, respectively) (**A** and **B**). In addition, Fra-1 was significantly higher expressed in triple-negative tumors compared with other groups (*p* = 0.039) (**C**). Gene expression (y-axis) was quantified by real-time PCR and normalized to UBC.

We classified the breast cancer tumors into different subtypes based on IHC staining as defined by O'Brien et al. [20]. ANOVA analysis revealed a significant difference among subtypes of breast cancer for Fra-1 expression (*p* = 0.039) (Table 3 and Figure 5C). Further analysis showed that Fra-1 mRNA expression was significantly higher in triple-negative tumors compared with luminal carcinomas (*p* = 0.01). However, the difference between the transcript levels in TNBC and HER2-enriched breast cancers was not statistically significant. When comparing the mRNA expression of AP-1 family members with known molecular markers of breast cancer, Fra-1 was significantly higher expressed in tumors classified as ERα negative tumors compared with ERα positive tumors (*p* = 0.012) (Figure 5A). Similarly, the expression level of Fra-1 was significantly higher in PR negative tumors compared with PR positive tumors (*p* = 0.037) (Figure 5B). Additionally, there was no correlation between the mRNA expression of other JUN and FOS family members and hormone receptor status.

**Table 3:** **Analyses of mRNA expression of AP-1 family members in relation to subtypes of breast cancer**

	Mean (±SD)			P Value (Among subtypes)
Variable	Luminal tumors	Triple-negative tumors	HER2-enriched tumors	
**c-Fos** mRNA Level	0.024 ± 0.038	0.012 ± 0.014	0.011 ± 0.009	0.834
**Fra-1** mRNA Level	0.024 ± 0.026	0.051 ± 0.043	0.026 ± 0.019	0.039^*^
**Fra-2** mRNA Level	0.330 ± 0.439	0.381 ± 0.253	0.197 ± 0.155	0.238
**Fos-B** mRNA Level	0.189 ± 0.283	0.090 ± 0.120	0.082 ± 0.108	0.214
**c-Jun** mRNA Level	0.076 ± 0.065	0.044 ± 0.027	0.068 ± 0.037	0.460
**Jun-B** mRNA Level	0.885 ± 0.612	1.327 ± 1.655	0.983 ± 0.695	0.708
**Jun-D** mRNA Level	0.475 ± 0.407	0.398 ± 0.235	0.490 ± 0.280	0.893

One-way ANOVA was used to calculate the p values among different subtypes. Fra-1 expression showed a significant difference among subtypes.

*Post-hoc analysis using LSD revealed that Fra-1 expression was significantly higher in triple-negative tumors compared with luminal carcinomas (*p* = 0.01). In addition, Fra-1 expression was marginally significantly higher in triple-negative tumors compared with HER2-enriched (*p* = 0.067).

### Association of AP-1 mRNA levels with clinical characteristics

We investigated the mRNA expression of AP-1 family members in relation to clinical characteristics such as clinical staging, histological grading, lymph node and menopausal statuses and age. We found significant associations between the expression of c-Fos and Fos-B with histological grading, with about 40 percent lower expression of c-Fos [Odds ratio (OR) = 0.62, 95% confidence interval (CI): 0.42 - 0.94] and Fos-B (OR = 0.62, 95% CI: 0.41 - 0.93) in grade two compared with grade one (Table 4). In addition, there were significant associations between the expressions of c-Fos (*p* = 0.02), Fra-1 (*p* = 0.05) and Jun-B (*p* < 0.0.1) with stage, where the lowest expression levels were observed for the combined stages III and IV compared with the other stages. Moreover, when comparing the mRNA expression of AP-1 family members according to menopausal status, we found a higher expression of Jun-D in premenopausal patients compared with postmenopausal patients (OR = 0.55, 95% CI: 0.30 - 0.99) (Table 5). Furthermore, we observed more than 50% lower expression of Fra-1 and Jun-B in tumors with lymph node involvement compared to tumors without lymph node involvement, where the ORs were 0.43 (95% CI: 0.22-0.83) and 0.43 (95% CI: 0.20-0.92), respectively (Tables 4 and 5). Additionally, since age might play a role as a potential confounder in the association of AP-1 mRNA levels with clinical characteristics, we correlated the mRNA expression levels of selected genes with age, and we found no correlation between mRNA expression levels of selected genes and age (data not shown).

**Table 4:** **ORs and 95% CIs for FOS member mRNA expression in tumors with clinicopathological parameters**

Parameter	OR (95% CI)			
	c-Fos	Fra-1	Fra-2	Fos-B
**Menopausal status**				
Pre-menopause	1	1	1	1
Post-menopause	0.95 (0.71-1.26)	1.24 (0.72-2.14)	0.78 (0.48-1.26)	0.84 (0.60-1.19)
P value	0.71	0.43	0.32	0.33
**Lymph node status**				
Negative	1	1	1	1
Positive	0.72 (0.50-1.06)	0.43 (0.22-0.83)	0.83 (0.49-1.41)	1.00 (0.70-1.46)
P value	0.09	0.01^*^	0.49	0.98
**Stage**				
I	1	1	1	1
II	1.18 (0.70-2.01)	0.71 (0.29-1.75)	0.83 (0.38-1.82)	1.02 (0.58-1.78)
III and IV	0.75 (0.44-1.26)	0.37 (0.14-0.99)	0.78 (0.35-1.76)	0.86 (0.48-1.53)
P value	0.02^*^	0.05^*^	0.84	0.64
**Grade**				
1	1	1	1	1
2	0.62 (0.42-0.94)	0.99 (0.56-1.76)	1.14 (0.62-1.90)	0.62 (0.41-0.93)
3	0.70 (0.37-1.29)	1.15 (0.42-3.17)	1.23 (0.50-3.04)	0.73 (0.37-1.44)
P value	0.04^*^	0.95	0.83	0.05^*^

ORs and CIs from logistic regression model, stratified on some parameters including menopausal and lymph node statuses, grade and stage.

*Abbreviations:* *OR*, Odds ratio; *CI*, Confidence interval.

^*^Statistically significant (*p* < 0.05 in two-tailed test).

**Table 5:** **ORs and 95% CIs for JUN member mRNA expression with clinicopathological parameters**

Parameter	OR (95% CI)		
	c-Jun	Jun-B	Jun-D
**Menopausal status**			
Pre-menopause	1	1	1
Post-menopause	0.80 (0.49-1.3)	0.77 (0.42-1.41)	0.55 (0.30-0.99)
P value	0.36	0.40	0.04^*^
**Lymph node status**			
Negative	1	1	1
Positive	0.84 (0.48-1.45)	0.43 (0.20-0.92)	0.95 (0.52-1.71)
P value	0.52	0.03^*^	0.86
**Stage**			
I	1	1	1
II	1.49 (0.67-3.30)	0.65 (0.22-1.97)	1.40 (0.59-3.33)
III and IV	0.881 (0.40-1.92)	0.25 (0.07-0.82)	0.93(0.39-2.23)
P value	0.14	< 0.01^**^	0.37
**Grade**			
1	1	1	1
2	0.66 (0.37-1.192)	0.56 (0.28-1.13)	1.11 (0.63-1.95)
3	0.54 (0.21-1.36)	0.65 (0.20-2.13)	0.98 (0.37-2.63)
P value	0.26	0.24	0.92

ORs and CIs from logistic regression model, stratified on parameters including menopausal and lymph node statuses, grade and stage.

*Abbreviations:* *OR*, Odds ratio; *CI*, Confidence interval.

^*^Statistically significant (*p* < 0.05 in two-tailed test).

^**^Statistically significant (*p* < 0.01 in two-tailed test).

## Discussion

In the present study, we conducted a comprehensive analysis of mRNA expression levels of AP-1 family members in 72 primary breast tumors and 37 adjacent tissues. However, this approach has its limitations when addressing the correlation of biomarkers with tumor development. Future studies could be designed to also allow sampling of normal breast tissue. However, also this approach has limitations because the percentage of epithelial cells is generally higher in breast cancer tissue. Analysis of samples after microdissection of the corresponding cell types from tumor and adjacent tissue as well as normal breast tissue would constitute an important development to identify tumor biomarkers.

The median age of the studied population at the time of diagnosis was 46.5 years. This is lower than the median age for breast cancer diagnosis worldwide [21]. The lower median age at diagnosis is consistent with the established younger age at diagnosis of breast cancer in developing countries [22, 23], which is believed to relate to the younger population in developing countries [24], which cause higher number of young patients.

In this study we observed that c-Fos and c-Jun were significantly lower expressed in tumors compared with adjacent tissues. Whereas, Fra-1, Fra-2, Jun-B and Jun-D were significantly higher expressed in tumors compared with adjacent tissue. Finally, our analysis suggests that two members of the AP-1 family members, Fra-1 and Jun-B are associated with clinical parameters and thus might provide novel markers for breast cancer. Taking into consideration the efficiency of the different PCR reactions, we estimate that across all samples, Jun B is expressed at the highest level followed by Jun-D. C-Jun and c-Fos and Fra-2 are expressed at about equal levels while Fos-B and Fra-1 are expressed at a lower level as determined from this analysis (Data not shown).

Consistent with previous reports, our results revealed that c-Fos and c-Jun are expressed at a higher level in adjacent tissues compared with tumors [13, 25]. Smith et al investigated the expression and activity of the AP-1 complex in human mammary epithelial cells (HMECs) at different stages including normal, immortal, oncogene-transformed and cancer. They showed that normal cells and immortal HMECs have higher mRNA and protein expression levels of c-Fos and c-Jun compared with human breast cancers [25].

Our results showing high expression of Fra-1 in breast tumors and its differential expression between ERα positive and ERα negative tumors are consistent with previous studies [14, 17, 26, 27]. Fra-1 was significantly higher expressed in triple-negative tumors compared with other groups (Figure 5C). However, the difference between the transcript levels in TNBC and HER2-enriched breast cancers was only marginally statistically significant (*p* = 0.067). Thus, Fra-1 would not appear to define an auxiliary diagnostic marker for TNBC, which is clearly need in light of the remarkable challenges that this breast cancer subtype presents to researchers and clinicians. *In vitro* studies have shown that Fra-1 expression is associated with cell motility, proliferation and invasiveness [28]. However, our findings showed that the expression of Fra-1 was significantly higher in nodal negative tumors compared with nodal positive tumors, and furthermore no correlation was observed between Fra-1 expression and metastasis status. In a cohort study Schroder et al. investigated Fra-2 mRNA expression in 167 patients and found significant correlations of high Fra-2 expression with younger age, nodal involvement, high grading and ER negative tumors [18]. However, our findings did not show any association of Fra-2 mRNA expression with ER or nodal status and grading.

Our results demonstrated that there was a significant association between increased Jun-B mRNA expression and reduced tumor size and tumor stage. Moreover, Jun-B mRNA expression was significantly higher in nodal negative tumors compared with nodal positive tumors. These findings imply that increased Jun-B expression could be related to a less aggressive tumor behavior. These results are similar with previous studies suggesting that Jun-B could have a role as a tumor suppressor [15].

Milde-Langosch et al. investigated Fos-B mRNA and protein expression in human breast tumors and normal breast tissues and showed that Fos-B was highly expressed in normal lobules and ducts with carcinomas frequently displaying loss of expression and weak immunostaining. They showed that Fos-B might be necessary for normal proliferation and differentiation of mammary epithelial cells and also Fos-B is down-regulated in poorly differentiated breast tumors [29]. Our results, displaying a marginally significant association between increased Fos-B mRNA expression and well-differentiated status are consistent with this report. In contrast, our findings did not show a significant difference in Fos-B expression between tumors and adjacent tissues.

There was no clear correlation between expression of AP-1 members in tumors versus surrounding tissues and the expression in higher grade and higher stage tumors. For example c-Fos was lower expressed in tumors versus surrounding tissues and the expression was also lower in higher grade and higher stage tumors while Jun-B expression was higher in tumors compared to surrounding tissues while the expression was lower in higher grade and stage tumors.

In this study we focus on the JUN and FOS family members of the AP-1 family of transcription factors. However additionally, members of the ATF (activating transcription factor) and MAF (musculoaponeurotic fibrosarcoma) protein families have been shown to contribute to this complex and could thus be considered as potential additional biomarkers in breast cancer.

## Conclusions

Overall, our findings reveal, among others, that Fra-1 mRNA levels are higher in ERα negative and PR negative breast cancer tumors. In addition, Jun-B overexpression has outstanding discrimination ability (AUC = 0.983) to differentiate tumor tissues from adjacent tissues. We therefore suggest that AP-1 family members should be further evaluated in larger cohorts as possible biomarkers in breast cancer.

## Electronic supplementary material

Additional file 1: Figure S1: Expression of AP-1 family members comparing tumor and adjacent tissues. The expression of Fra-1 (B), Fra-2 (C), Jun-B (F) and Jun-D (G) are significantly higher in tumors compared with adjacent tissues (*p* < 0.001), whereas the expression of c-Fos (A) and c-Jun (E) are significantly lower in tumors compared with adjacent tissues (*p* < 0.001). A paired model was applied. Gene expression (y-axis) was quantified by real-time PCR and normalized to UBC. **Figure S2**. mRNA levels of ERα display a positive correlation with mRNA levels of Jun-B and Jun-D. mRNA levels of ERα displayed a significant positive correlation with mRNA levels of Jun-B and Jun-D among ERα positive tumors (n = 47). ERα, Jun-B and Jun-D mRNA levels were quantified by real-time PCR and normalized to UBC. (DOCX 397 KB)

## Authors’ original submitted files for images

Below are the links to the authors’ original submitted files for images. Authors’ original file for figure 1
Authors’ original file for figure 2
Authors’ original file for figure 3
Authors’ original file for figure 4
Authors’ original file for figure 5

## Abbreviations

AP-1: Activator protein-1
ER: Estrogen receptor
PR: Progesterone receptor
HER2: Human epithermal growth factor receptor 2
UBC: Ubiquitin C
mRNA: Messenger ribonucleic acid
cDNA: Complementary deoxyribonucleic acid
Fra-1: Fos-related antigen 1
Fra-2: Fos-related antigen 2
RT-qPCR: Reverse transcriptase quantitative polymerase chain reaction
SBR grading system: Scarff, Bloom, Richardson, grading system
ROC: Receiving operating characteristic
AUC: Area under the curve
OR: Odds ratio
CI: Confidence interval
HMECs: Human mammary epithelial cells
ATF: Activating transcription factor
MAF: Musculoaponeurotic fibrosarcoma.
