# Supplementary figures and images for: Expression of activator protein-1 (AP-1) family members in breast cancer
Source: BMC Cancer. 2013 Sep 28;13:441. doi: 10.1186/1471-2407-13-441 (PMC3849565; doi:10.1186/1471-2407-13-441)

**Supplementary figures**

**Figure 1**


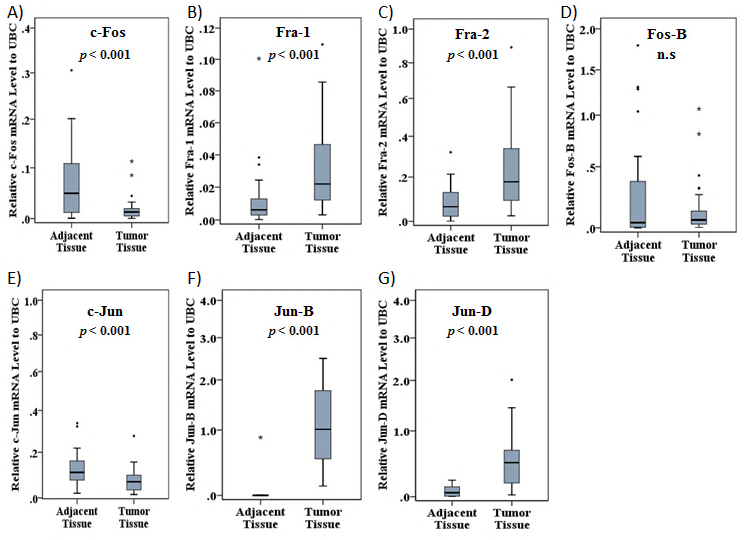


**Figure 2**

**
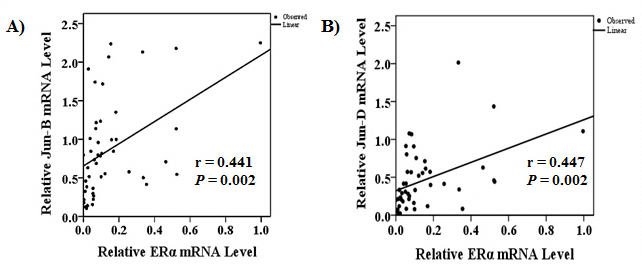
**

Supplement: Supplementary file 1 — Additional file 1: Figure S1: Expression of AP-1 family members comparing tumor and adjacent tissues. The expression of Fra-1 (B), Fra-2 (C), Jun-B (F) and Jun-D (G) are significantly higher in tumors compared with adjacent tissues (p < 0.001), whereas the expression of c-Fos (A) and c-Jun (E) are significantly lower in tumors compared with adjacent tissues (p < 0.001). A paired model was applied. Gene expression (y-axis) was quantified by real-time PCR and normalized to UBC. Figure S2. mRNA levels of ERα display a positive correlation with mRNA levels of Jun-B and Jun-D. mRNA levels of ERα displayed a significant positive correlation with mRNA levels of Jun-B and Jun-D among ERα positive tumors (n = 47). ERα, Jun-B and Jun-D mRNA levels were quantified by real-time PCR and normalized to UBC. (DOCX 397 KB) [file 12885_2013_4091_MOESM1_ESM.docx]

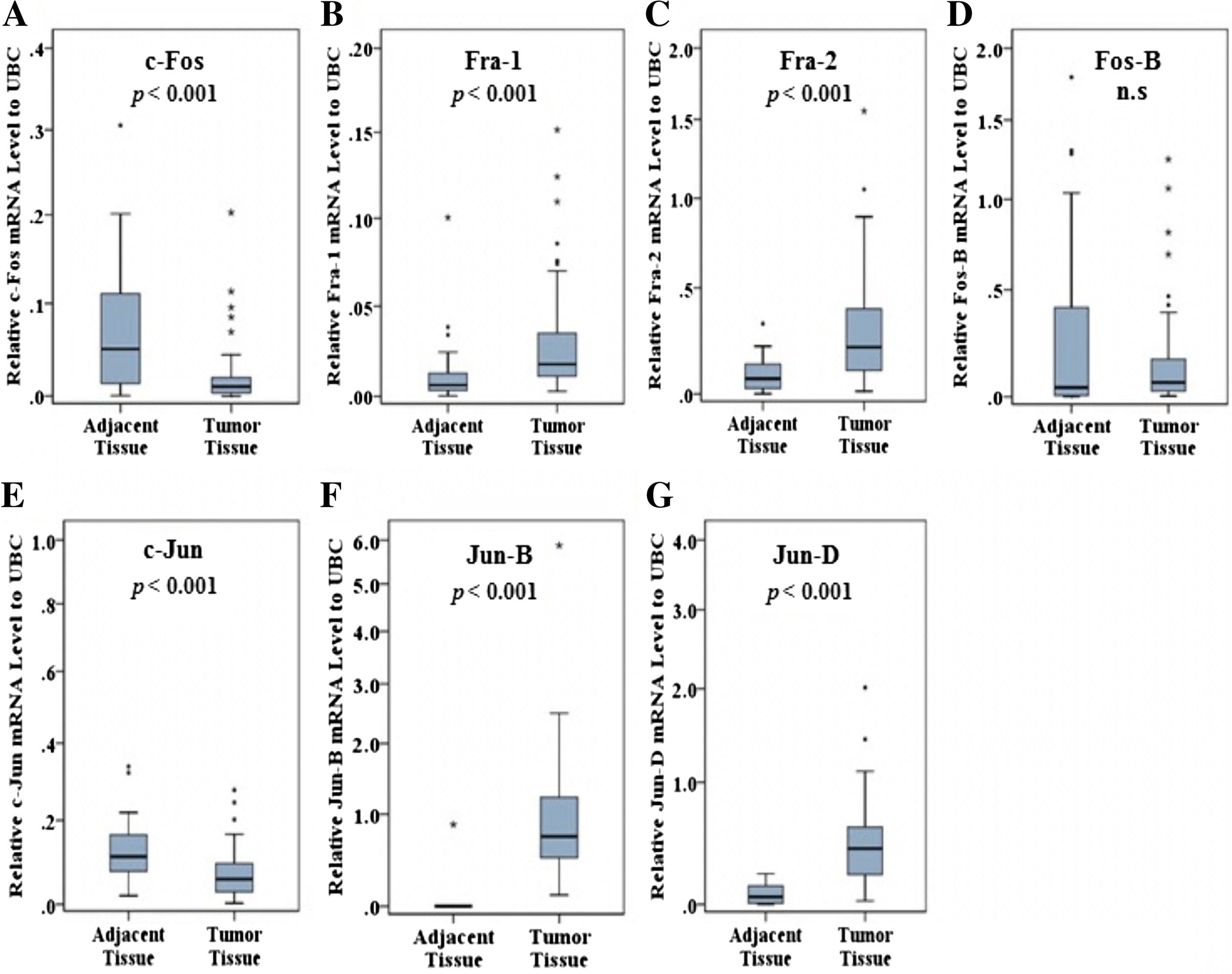

Supplement: Supplementary file 2 — Authors’ original file for figure 1 [file 12885_2013_4091_MOESM2_ESM.tiff]

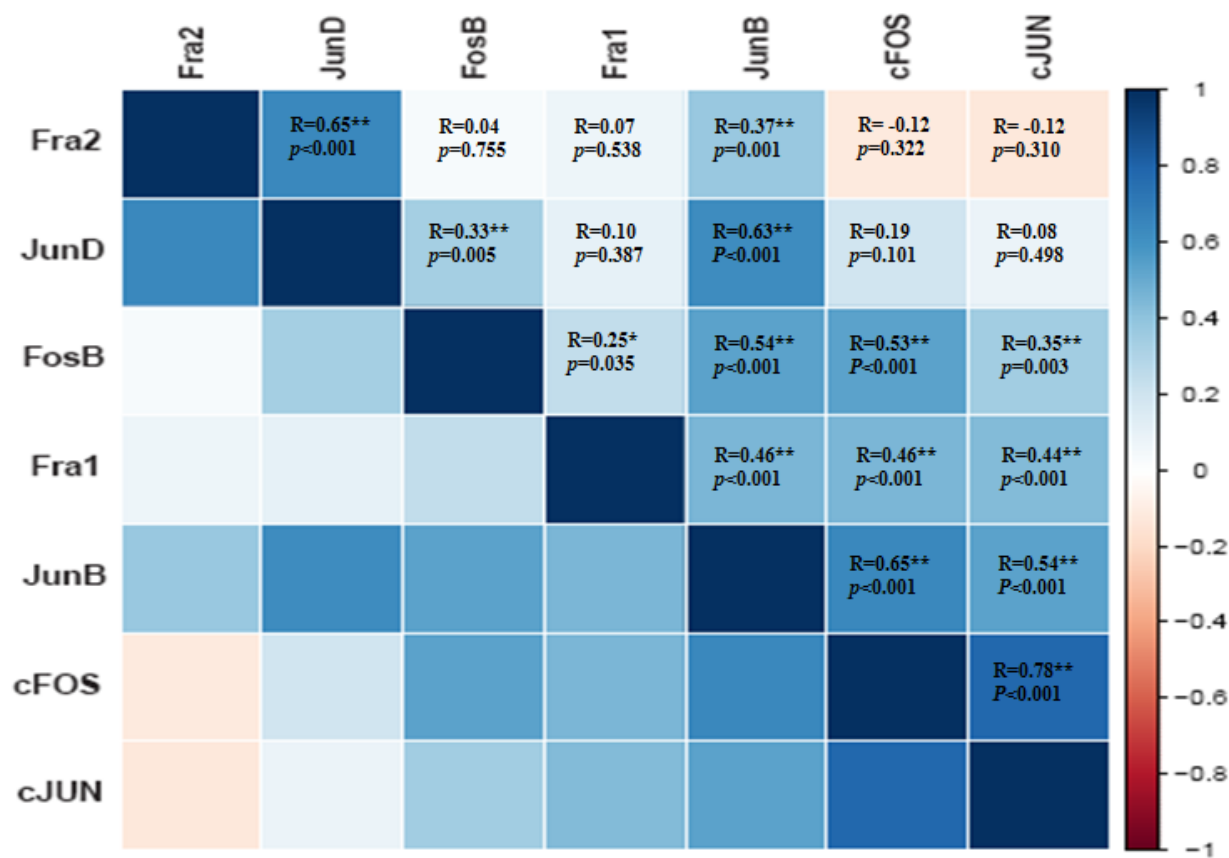

Supplement: Supplementary file 4 — Authors’ original file for figure 3 [file 12885_2013_4091_MOESM4_ESM.pdf]

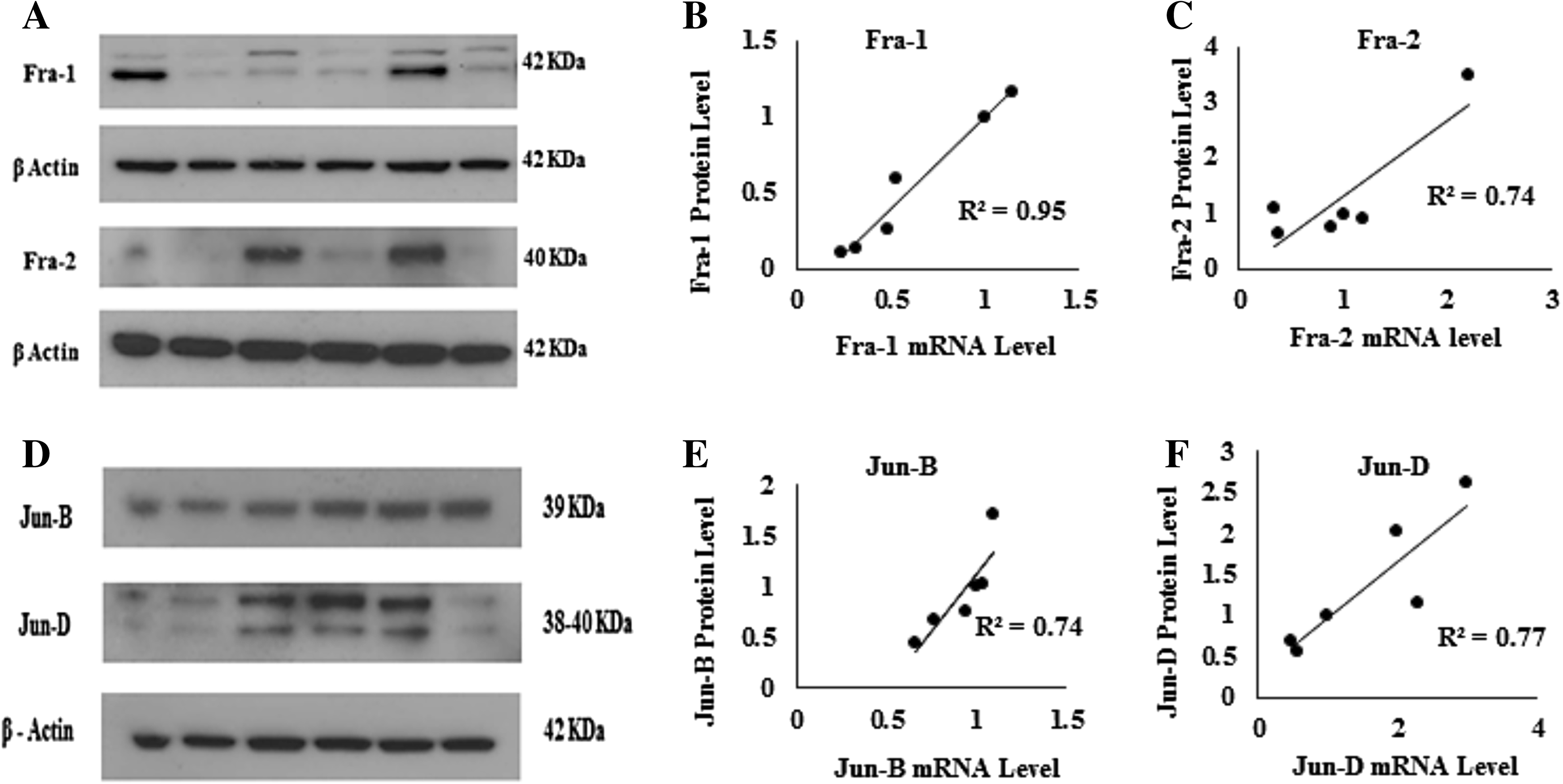

Supplement: Supplementary file 5 — Authors’ original file for figure 4 [file 12885_2013_4091_MOESM5_ESM.tiff]

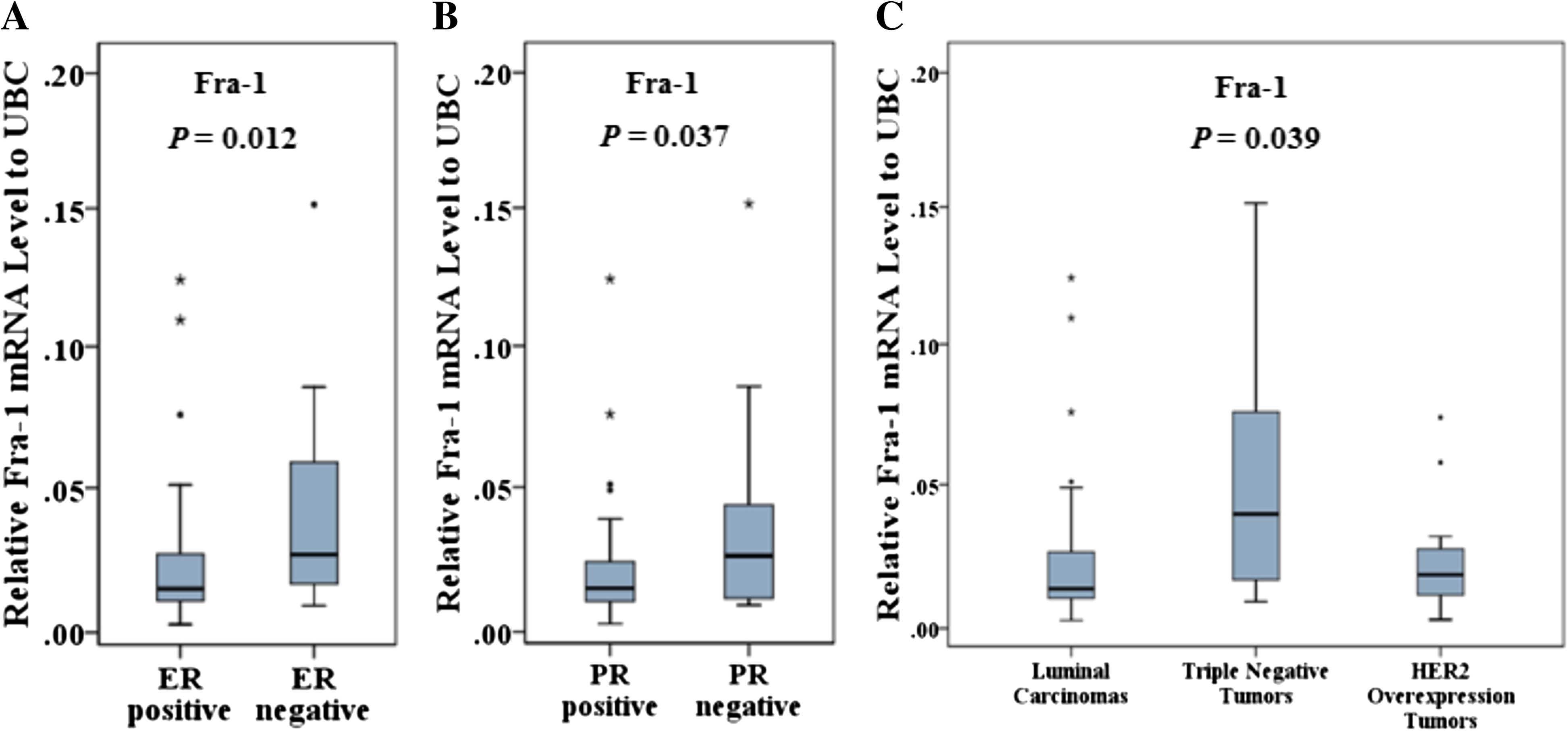

Supplement: Supplementary file 6 — Authors’ original file for figure 5 [file 12885_2013_4091_MOESM6_ESM.tiff]
