# Supplementary material for: Expression of activator protein-1 (AP-1) family members in breast cancer
Source: BMC Cancer. 2013 Sep 28;13:441. doi: 10.1186/1471-2407-13-441 (PMC3849565; doi:10.1186/1471-2407-13-441)
Supplement: Supplementary file 3 — Authors’ original file for figure 2 [file 12885_2013_4091_MOESM3_ESM.pdf]

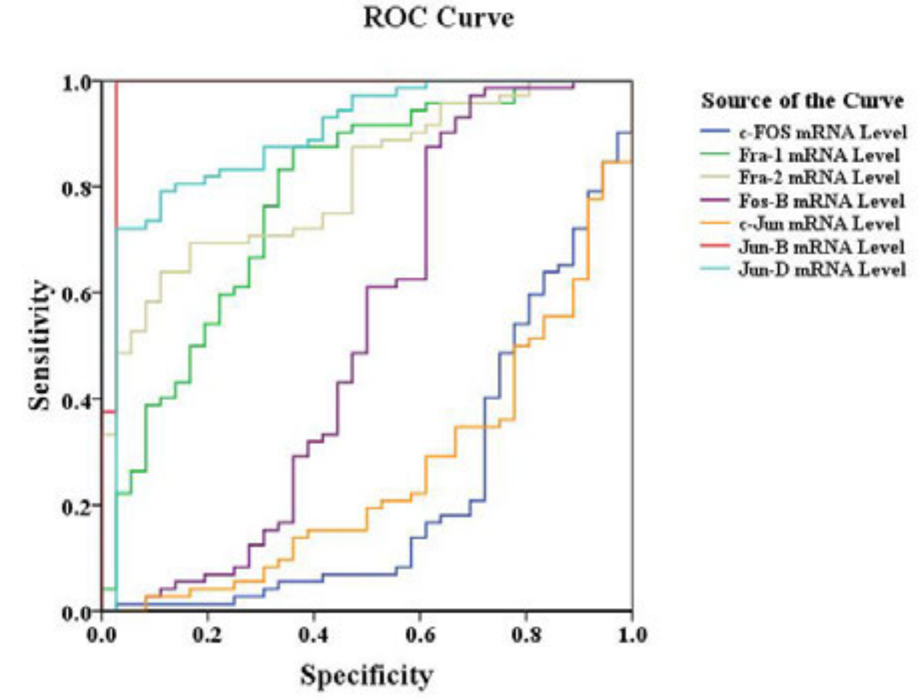
ROC Curve
Sensitivity
0.0
0.2
0.4
0.6
0.8
1.0
Specificity
Source of the Curve
c-FOS mRNA Level
Fra-1 mRNA Level
Fra-2 mRNA Level
Fos-B mRNA Level
c-Jun mRNA Level
Jun-B mRNA Level
Jun-D mRNA Level
